# Validated Simultaneous Gradient Ultra-Performance Liquid Chromatographic Quantification of Some Proton Pump Inhibitor Drug Residues in Saudi Pharmaceutical Industrial Wastewater

**DOI:** 10.3390/molecules26144358

**Published:** 2021-07-19

**Authors:** Sherif A. Abdel-Gawad, Hany H. Arab, Alhumaidi B. Alabbas

**Affiliations:** 1 Pharmaceutical Chemistry Department, College of Pharmacy, Prince Sattam Bin Abdulaziz University, Al Kharj 11942, Saudi Arabia; ab.alabbas@psau.edu.sa; 2 Analytical Chemistry Department, Faculty of Pharmacy, Cairo University, Cairo ET-11562, Egypt; 3 Department of Pharmacology and Toxicology, College of Pharmacy, Taif University, P.O. Box 11099, Taif 21944, Saudi Arabia; h.arab@tu.edu.sa

**Keywords:** drug residues, environmental, proton pump inhibitors, UPLC, wastewater

## Abstract

Monitoring and quantification of active pharmaceutical ingredients (APIs) in the environment constitute important and challenging tasks, as they are directly associated with human health. Three commonly used proton pump inhibitors (PPIs), namely, omeprazole sodium (OMP), pantoprazole sodium (PNT), and lansoprazole sodium (LNZ) are well separated and quantified using ultra-performance liquid chromatography (UPLC) in pharmaceutical industrial wastewater. The separation of the studied drugs was performed on a stationary phase with a Waters^TM^ column (100 × 2.1 mm, 1.7 µm). The mobile phase was composed of methanol:0.05 M potassium dihydrogen phosphate buffer (adjusted to pH 7.5 using NaOH) (50:50, *v*/*v*). The elution process was done in gradient mode by changing the relative proportions of the mobile phase components with time to get an optimum separation pattern. The flow rate of the developing system was adjusted to 0.8 mL/minute. Detection of the separated drugs was performed at 230 nm. The studied drugs were quantified in the concentration range of 10–200 ng/mL for all drugs. The cited method was fully validated according to the international conference on harmonization (ICH-Q2B) guidelines, then it was applied successfully for quantification of the studied PPIs in real wastewater samples after their solid phase extraction (SPE).

## 1. Introduction

Stomach acid is a natural and valuable chemical contributor to the digestion process. However, in excess or in the wrong place it is a menace, inflaming and irritating the esophagus. Additionally, it may cause heartburn and sometimes contribute to the development of ulcers in the stomach, the duodenum, and the first part of the small intestine. Proton pump inhibitors (PPIs) are a group of medications whose main action is a pronounced and long-lasting reduction in stomach acid production. They are widely prescribed for the treatment of gastroesophageal reflux disease (GERD), acid reflux, heartburn, and stomach ulcers. Additionally, they are considered an important component in the triple therapy of *Helicobacter pylori* infection [1].

In the last few years, the widespread use of PPIs may have caused their existence in the environment, especially in surface water. Despite many studies having carried out progressive determinations of pharmaceutical compounds in environmental wastewaters and their negative influences on human health, there is no limit value for the presence of these pollutants in soil or natural water [2]. PPIs may cause a lot of adverse effects when reaching the human body. These adverse effects may be mild like headaches, diarrhea, constipation, abdominal pain, flatulence, and fever. Long term exposure to PPIs may lead to serious effects, including an increased risk of *Clostridium difficile* infection in the colon, gastric carcinoid tumor, and osteoporosis-related fractures of the hip, wrist, or spine. Additionally, the increased risk of heart attacks may occur due to long-term exposure to PPIs. The residues of PPIs may reach children from the surrounding environment and may cause serious effects upon long-term exposure. These effects are the increased risk of gastrointestinal and respiratory tract infections, vitamin B12 deficiency, hypomagnesemia, and bone fracture. PPIs are excreted from the body through the urine, which could reach the hospital sewage. Additionally, the studied drugs could be present in the wastewater of pharmaceutical plants and so they could reach surface water easily. Due to the negative influences of these compounds on human health upon long-term exposure, they should not be present in surface water or soil even at very low concentrations. They are regarded as pollutants [3].

Omeprazole sodium (OMP), pantoprazole sodium (PNT), and lansoprazole sodium (LNZ) are three commonly used PPIs in the Saudi market. They are included in this study to monitor their amounts in industrial wastewater.

OMP (C_17_H_18_N_3_NaO_3_S), sodium;5-methoxy-2-[(4-methoxy-3,5-dimethyl pyridine-2-yl)methylsulfinyl]benzimidazol-1-ide, decreases the capability of the parietal cells to secrete gastric acid through its action on the (H^+^/K^+^)-ATPase enzyme [4]. Many techniques have been applied for the quantification of OMP in different samples. It was quantified spectrophotometrically depending on its combination with ion-pairing agents, like bromophenol blue and orange G, then the formed reaction products were measured at 408 nm and 508 nm, respectively [5]. OMP was determined simultaneously with diclofenac potassium [6] or aspirin [7] by using either the simultaneous equation method or zero-order and area under curve methods. High-performance liquid chromatography (HPLC) was applied for the determination of OMP in capsules depending on its separation from the C18 column and UV-detection [4]. Additionally, it was quantified in plasma after its extraction with tertiary butyl methyl ether, separation on a C18 column, and detection using a photodiode array detector [8]. Liquid chromatography coupled with tandem mass spectroscopy (LC-MS/MS) was applied for the determination of OMP in human plasma. It was detected using multiple reaction monitoring (MRM) mode [9]. Ultra-performance liquid chromatography (UPLC) was applied for the determination of OMP, either simultaneously with domperidone in capsules [10] or with its related substances using TOF/MS detection [11]. Meanwhile, UPLC was used for OMP assay in its magnesium blend [12].

PNT (C_16_H_14_F_2_N_3_NaO_4_S), sodium;5-(difluoromethoxy)-2-[(3,4-dimethoxypyridin-2-yl)methylsulfinyl]benzimidazol-1-ide, was quantified titrimetrically and spectrophotometrically in pharmaceuticals using cerium (IV) sulphate as an oxidimetric agent [13]. It was also determined spectrophotometrically with OMP through complex formation with iron (III) in an aqueous ethanol medium [14]. LC-MS/MS was applied for the determination of PNT using LNZ as an internal standard (IS), either in human plasma [15] or in human urine [16]. The same technique was also applied for the sensitive monitoring of the studied drug in a bioequivalence study [17].

LNZ (C_16_H_13_F_3_N_3_NaO_2_S), sodium;2-[[3-methyl-4-(2,2,2-trifluoromethoxy)pyridin-2-yl]methylsulfinyl]benzimidazol-1-ide, is a potent PPI. It was determined spectrophotometrically in capsules and spiked human urine through ion-pair complex formation with bromocresol purple and bromothymol blue [18]. Kinetic spectrophotometry was applied for the quantification of LNZ in a pharmaceutical formulation *via* its reaction with alkaline potassium permanganate at room temperature [19]. Extractive spectroscopy was applied for the determination of LNZ in pharmaceutical dosage form after its reaction with metanil yellow and methyl orange followed by extraction with chloroform [20]. HPLC was applied for the determination of LNZ with UV-detection [21]. Additionally, the same technique was applied for the quantification of the studied drug with its related impurities [22]. UPLC was applied for the determination of LNZ in commercial pharmaceutical products [23]. Additionally, the same technique was demonstrated for the simultaneous quantification of the studied drug with naproxen [24]. LC-MS/MS was applied for the determination of LNZ in human plasma, using esomeprazole as an IS [25].

In the last few years, there has been an enormous increase in *Helicobacter pylori* infections all over the world. This, in turn, has increased the prescribing rates of PPIs, especially in the Saudi market. OMP, PNT, and LNZ are the most widely and frequently prescribed PPIs in the Saudi market. Many research papers have been published dealing with different pharmaceuticals, including PPIs in the aquatic environment for many regions in the world, especially in Europe [26,27,28,29]. OMP, PNT, and LNZ are manufactured by many factories in the Kingdom of Saudi Arabia (KSA). Therefore, there is a big chance for these drugs to reach surface and irrigation water. Accordingly, there is an imminent threat to human health, especially in the regions surrounding the manufacturing factories. Therefore, there is an urgent need to optimize, validate, and apply an analytical method to monitor and quantify these three PPIs in industrial wastewater for that pharmaceutical factory.

UPLC is a chromatographic system that can use a combination of reversed-phase packing substances with a size of 1.7 μm and an operation pressure in the 6000–15,000 psi range. It has many advantages over conventional HPLC, including a greater sensitivity as characterized by a greater signal to noise (S/N) ratio due to the reduction in zone broadening. Additionally, it is characterized by better chromatographic peak resolution and increased speed of analysis. UPLC is characterized by higher sensitivity and selectivity compared to the molecular spectrophotometric methods. Regarding the suggested technique (UPLC), when compared to LC-MS/MS it has comparable sensitivity but with more economic operating expenses. Additionally, UPLC does not require special instrument assembly [30]. After an extensive literature review, no published papers have directly conducted a UPLC determination of the studied PPI drug residues in Saudi pharmaceutical industrial wastewater.

The main goal of this work is to optimize and validate a simple, sensitive, accurate, and reproducible UPLC method to quantify the studied drugs in Saudi pharmaceutical industrial wastewater after their pretreatment using solid-phase extraction (SPE).

## 2. Results

This work demonstrates the capability of selective simultaneous quantification of three commonly used PPI drug residues in pharmaceutical wastewater samples using UPLC in wastewater samples, which in turn, make the environmental monitoring task of the studied drugs, an easy process.

### 2.1. Method Optimization

**Figure 1:**
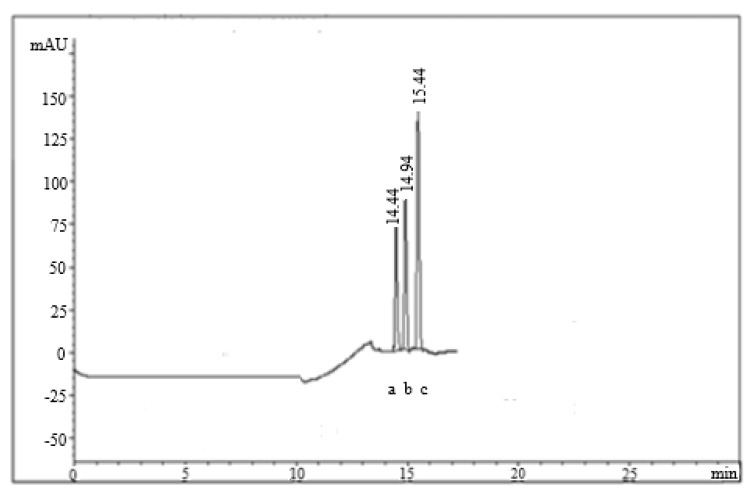
UPL chromatogram for the separation pattern of OMP (**a**), PNT (**b**), and LNZ (**c**) reference materials.

The best separation pattern was achieved using the stationary phase of the Waters^TM^ column (100 × 2.1 mm, 1.7 µm) and a mobile phase composed of methanol:0.05 M potassium dihydrogen phosphate buffer (adjusted to pH 7.5 using NaOH) (50:50, *v*/*v*). The composition of the mobile phase was changed by increasing the percentage of methanol by 5% and decreasing the percentage of the buffer by 5% every 4 min. The mobile phase flow rate was adjusted to 0.8 mL/min. On the other hand, detection of eluted drugs was performed at 230 nm. The retention times were 14.40 min for OMP, 14.90 min for PNT and 15.40 min for LNZ, Figure 1.

Acceptable system suitability parameters were obtained in Table 1. The values for the number of theoretical plates (N) and height equivalent to theoretical plates (HETP) confirmed excellent column efficiency. At the same time, good selectivity and resolution were ensured by the resolution factor (Rs). Additionally, the tailing factor value confirmed acceptable peak symmetry.

### 2.2. Method Validation

The validation was carried out according to ICH-Q2B guidelines [31]. A linear relation was obtained between the peak area and the concentration of the studied drugs in the concentration range 10–200 ng/mL. The following regression equations were computed to be:

PA (OMP) = 97.116 C + 1.4526 r = 0.9999(1)

PA (PPZ) = 52.406 C + 9.1226 r = 0.9998(2)

PA (LNZ) = 94.542 C + 7.1121 r = 0.9997(3)

where, PA: peak area. C: Concentration (ng/ mL). r: Correlation coefficient.

The validation sheet showed acceptable accuracy, repeatability, and intermediate precision as demonstrated in Table 2. Assessment of the method’s robustness was also done by carrying out a slight change in the composition of the mobile phase, pH, and flow rate. All the results confirmed that there is no significant effect on the proposed method when carrying out these slight changes, which confirmed the robustness of the suggested method. The calculated values of LOD and LOQ reflected the acceptable sensitivity of the cited method.

### 2.3. Method Application

The cited method was effectively tested for quantification of the cited drugs by spiking them in distilled and tap water samples followed by the application of the optimized techniques of sample preparation and quantification. The results are given in Table 3.

**Figure 2:**
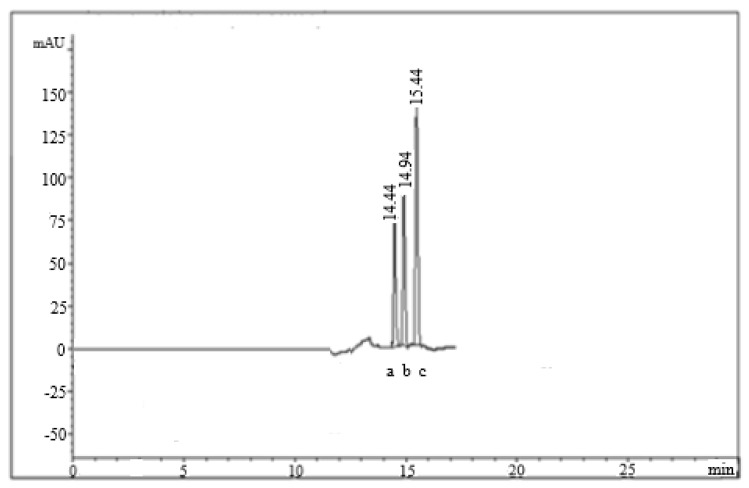
UPL chromatogram for the separation pattern of OMP (**a**), PNT (**b**), and LNZ (**c**) in actual wastewater sample.

Additionally, actual wastewater samples were carefully treated using the optimized sample preparation technique, then they were chromatographed by applying the optimized chromatographic parameters (Figure 2). The results were compared to those obtained by analyzing the actual wastewater samples using reference methods for the determination of OMP, PNT, and LNZ [9,17,25] after sample pretreatment by the same sample preparation protocol (Table 4). Method performance for the determination of the cited drugs was also validated by the application of the standard addition procedure. The recovery % was calculated for the added pure PPI concentrations (Table 5).

## 3. Discussion

There is no doubt that monitoring environmental pollutants is a crucial task for conserving human health. Pharmaceutical drugs reaching domestic water or plants can constitute a great danger to human health as they can expose the human body for a long time, which may lead to serious effects on the human body. The present work demonstrated the capability of the quantification of PPI residues in wastewater coming from the pharmaceutical industry using a simple and sensitive UPLC method to monitor the environmental levels of the studied drugs to avoid their harmful effects on the human body.

Method optimization was done to get the best separation pattern and optimum peak resolution. This task was confirmed via the introduction of a full system suitability scheme (Table 1). The capacity factor values for the studied PPIs ensured that there was adequate time for the studied drugs to interact with the stationary phase properly. The values of resolution factors ensured baseline to baseline peak separation and excellent peak resolution. At the same time, the values of N and HETP declared high column efficiency. The tailing factors’ values showed acceptable peak symmetry.

Method validation parameters were calculated and presented in a full validation sheet (Table 2). It confirmed the high accuracy and precision of the suggested method. Additionally, the validation sheet declared excellent method robustness that ensured the lack of considerable effects of slight changes in mobile phase composition, pH, and flow rate on the cited method performance. Meanwhile, an acceptable method sensitivity was declared by the linearity range, LOD, and LOQ. They were very suitable for the determination of the cited PPIs in the concentration levels supposed to be present in the actual wastewater samples coming from pharmaceutical industrial factories around the KSA.

The proposed sample preparation technique and the quantification procedure were successfully applied for the determination of the studied PPIs in both spiked distilled and tap water samples giving acceptable results that confirmed the efficiency of the sample preparation procedure and the accuracy of the suggested method (Table 3). The suggested method was also applied for the quantification of the studied drugs in actual wastewater samples obtained from different pharmaceutical industrial factories around the KSA. To validate the results obtained by the cited method, the found concentrations in the actual wastewater samples were compared with those obtained by applying the reference methods for the determination of the studied PPIs in the same samples [10,18,26], declaring good matching between the results (Table 4). The standard addition technique was well applied for the actual wastewater samples, confirming the excellent performance of the cited method (Table 5). The monitoring of the studied drugs by the developed method was found to be simple, economic, and suitable for environmental analysis.

## 4. Materials and Methods

### 4.1. Instruments

Ultra-performance liquid chromatography was done with a Waters^TM^ Acquity system (Milford, CT, USA) with column dimensions 100 × 2.1 mm, 1.7 µm. The detector was a UV-visible wavelength detector (Waters, 2489, Milford, CT, USA). SPE was performed using Agilent Bondesil ODS manually packed cartridges (Santa Clara, Canada).

### 4.2. Chemicals and Reagents

OMP, PNT, and LNZ bulk powder were kindly supplied by Riyadh Pharma (Riyadh, Kingdom of Saudi Arabia) and their percentage purities were labeled as 100.16 ± 0.36, 100.17 ± 0.41, and 99.45 ± 0.78, respectively. Methanol (HPLC-grade, purity ≥ 99.9%), acetonitrile (HPLC-grade, purity ≥ 99.9%), and distilled water (HPLC-grade) were purchased from Sigma-Aldrich (St. Louis, MO, USA). Potassium dihydrogen orthophosphate (HPLC-grade) was supplied by Fischer Chemicals™ (Zürich, Switzerland). Sodium hydroxide solution (HPLC-grade, LiChropur™, 49.0–51.0%) was purchased from Merck (Darmstadt, Germany).

### 4.3. Standard Solutions

Stock standard solutions of OMP, PNT, and LNZ (100 µg/mL) were prepared by accurately and separately weighing 10 mg of each bulk powder into separate 100-mL volumetric flasks. The powder was dissolved in each flask, and the volume was brought to 100 mL using methanol. Working solutions of the studied drugs (1 µg/mL) were prepared from stock standard solutions (100 µg/mL) by diluting 1 mL of each stock standard solution (100 µg/mL) to 100 mL using methanol as a diluting solvent.

### 4.4. Method Optimization

Different stationary phases and mobile phases were tried to ensure good system suitability parameters.

### 4.5. Method Validation

The validation process was conducted according to the ICH-Q2B guiding protocol.

#### 4.5.1. Linearity

Different aliquots (1–20 µg) of the studied drugs were accurately and separately taken to a group of volumetric flasks (100-mL capacity). The volume of each flask was then completed with methanol to obtain a concentration of 10–200 ng/mL of each studied drug. Samples were then chromatographed using a Waters^TM^ column (100 × 2.1 mm, 1.7 µm, Milford, CT, USA). The mobile phase was methanol:0.05 M potassium dihydrogen phosphate buffer (adjusted to pH 7.5 using NaOH) (50:50, *v*/*v*). The composition of the mobile phase was changed by increasing the percentage of methanol by 5% and decreasing the percentage of the buffer by 5% every 4 min. A flow rate of 0.8 mL/min was maintained during the whole chromatographic analysis. The detection was done at 230 nm. Peak areas were plotted against concentration to get standard curves then the regression equations were computed.

#### 4.5.2. Accuracy

The accuracy can be defined as the percent of the recovered analyte from a definite quantity [31]. Results coming from nine samples of concentrations (50, 60, and 70 ng/mL of each studied drug) were analyzed using the procedure under linearity.

#### 4.5.3. Precision

It can be expressed as inter and intra-day precision, which is reported as % relative standard deviation for several experiments that are statistically significant so three concentrations of each studied drug (50, 60, and 70 ng/mL) were analyzed three times within the same day (intra-day) or on three successive days (inter-day) then the results were documented as %RSD.

#### 4.5.4. Detection and Quantification Limits

These can give us an idea about the method sensitivity, where LOD is the lowest detectable concentration and LOQ is the minimum quantified concentration. They were determined mathematically [31].

#### 4.5.5. Robustness

The robustness can be assessed by evaluating the effect of minor changes on the proposed procedure. This was done by changing the developing system composition by adding 1% acetonitrile to the used mobile phase. Additionally, the rate of flow varied by ±0.1 mL/min.

#### 4.5.6. System Suitability

A full system suitability protocol was prepared including different parameters indicating migration rates of the separated drugs (capacity factor), selectivity and resolution (resolution factors), column efficiency (N and HETP), and peak symmetry (tailing factor).

### 4.6. Method Application

#### 4.6.1. Wastewater Sample Collection and Storage

Wastewater samples were collected from different pharmaceutical industry sites around the KSA. Filtration of the samples was done using membrane filters made of nylon. The samples were then stored in a cool and dark place.

#### 4.6.2. Preparation of Actual Wastewater Samples

The actual wastewater samples were treated by SPE. First, the cartridges’ packing material was treated with six mL methanol and three mL water for pre-conditioning purposes. The samples were then vortex mixed for approximately 10 s to ensure sample homogeneity. The type and volume of eluting solvent and flow rate were optimized. A sample volume of 4 mL was loaded, followed by washing using 1 mL water twice to remove the unbound substances in each cartridge and reduce any interfering band in chromatograms. Finally, the studied drugs were eluted from the cartridge with 2 mL methanol. Twenty μL of each studied drug were then chromatographed.

#### 4.6.3. Determination of OMP, PNT and LNZ in Spiked Water Samples

The extraction procedure was evaluated by spiking distilled and tap water samples with different amounts of the studied drugs to get concentrations of 30, 40, and 50 ng/mL of the studied PPIs. The spiked samples were subjected to the extraction protocol then the extracted samples were analyzed using the optimized chromatographic technique.

#### 4.6.4. Determination of OMP, PNT and LNZ in Actual Wastewater Samples

Five actual wastewater samples were subjected to the optimized SPE procedure, then they were chromatographed following the optimized chromatographic conditions. Calibration plots were used to get the unknown concentrations. The obtained results were compared to those obtained by applying reference methods for the determination of OMP, PNT, and LNZ [10,18,26] after sample pretreatment by the same extraction protocol. Additionally, the suggested method was validated by the application of the standard addition procedure to the actual wastewater samples at three concentration levels (10, 20, and 30 ng/mL). The recovery % was calculated for the added pure PPI concentrations.

## 5. Conclusions

This work represents the optimization, validation, and application of UPLC method for the simple, sensitive, and accurate determination of OMP, PNT, and LNZ drug residues in Saudi pharmaceutical industrial wastewater. The cited method offers distinct advantages over the already published work. It has greater sensitivity and better peak resolution if compared to the conventional HPLC methods. Regarding its comparison with the LC-MS/MS methods, the suggested method is more economic and does not need a special instrument assembly. The suggested method has critical importance in tracing, detecting, and quantification of the studied PPIs drug residues in the pharmaceutical wastewaters, where their presence may lead to adverse consequences on human health and society.

## Figures and Tables

**Table 1:** Established system suitability parameters of the UPLC-method.

Parameter	OMP	PPZ	LNZ
t _R (min.)_^†^ (*n* = 3)	14.40 ± 0.15	14.90 ± 0.21	15.40 ± 0.17
Capacity factor (K)	71	74	76
Resolution factor (Rs)	-	5	5
Number of theoretical plates (N)	331,776	355,216	379,456
HETP *	3.1 × 10^−5^	2.8 × 10^−5^	2.6 × 10^−5^
Tailing factor (T)	1.04	1.04	1.04

^†^ Triplicate runs per sample. * Height equivalent to theoretical plates.

**Table 2:** Validation results of the proposed UPLC method.

Parameter	OMP	PPZ	LNZ
Accuracy (Mean * ± SD)	99.65 ± 1.21	102.65 ± 1.32	101.32 ± 0.95
Precision:			
Repeatability *	101.23 ± 0.72	99.79 ± 0.86	101.44 ± 1.15
Intermediate precision *	99.13 ± 0.84	100.34 ± 0.61	100.67 ± 0.79
**Robustness:**			
Mobile phase composition change	101.41 ± 0.85	99.26 ± 0.89	100.79 ± 0.92
pH change	99.76 ± 0.88	101.31 ± 1.38	101.22 ± 0.95
Flow rate change	101.95 ± 1.34	101.46 ± 1.18	99.57 ± 0.89
**Linearity:**			
Range (ng/mL)	10–200	10–200	10–200
Slope	97.116	52.406	94.542
Intercept	1.4526	9.1226	7.1121
Correlation coefficient (r)	0.9999	0.9998	0.9997
LOD (ng/mL)	2	2	2
LOQ (ng/mL)	10	10	10

* Average of three readings.

**Table 3:** Determination of OMP, PPZ, and LNZ in spiked water samples using the proposed method.

Specimen	OMP	PPZ	LNZ
Distilled water (Rec. % ± SD) *	102.52 ± 0.79	99.44 ± 1.14	99.98 ± 0.92
Tap water (Rec. % ± SD) *	101.04 ± 0.88	102.78 ± 1.34	101.69 ± 0.97

* Average of five measurements.

**Table 4:** Determination of OMP, PPZ, and LNZ in actual wastewater samples from industrial pharmaceutical plants.

Sample Number	OMP *		PPZ *		LNZ *	
	UPLC-Method	Reference Method [9]	UPLC-Method	Reference Method [17]	UPLC-Method	Reference Method [25]
Sample 1	20.02	19.98	30.55	30.59	15.72	15.43
Sample 2	25.23	25.23	21.13	21.77	31.79	31.69
Sample 3	31.67	31.89	20.56	20.91	40.01	39.99
Sample 4	39.87	39.58	40.56	40.22	21.68	21.59
Sample 5	22.97	22.46	25.37	25.12	41.83	41.11

* Concentrations are calculated in nanograms per milliliter.

**Table 5:** Application of standard addition procedure for PPIs determination.

Added PPIs (ng/mL)	OMP	PPZ	LNZ
	(Recovery % ± SD) *	(Recovery % ± SD) *	(Recovery % ± SD) *
10	101.67 ± 0.523	99.67 ± 0.652	100.89 ± 0.452
20	98.45 ± 0.967	102.67 ± 1.328	99.23 ± 0.891
30	99.89 ± 0.698	101.67 ± 0.945	101.12 ± 0.723

* Average of five readings.

## Data Availability

The datasets used and/or analyzed during the current study are available from the corresponding author on reasonable request.

## Sample Availability

The datasets used and/or analyzed during the current study are available from the corresponding author on reasonable request.
